# Decoding Sepsis: Unraveling Key Signaling Pathways for Targeted Therapies

**DOI:** 10.34133/research.0811

**Published:** 2025-10-01

**Authors:** Lingxuan Tang, Wangzheqi Zhang, Yan Liao, Weijie Wang, Yuxian Wu, Zui Zou, Changli Wang

**Affiliations:** ^1^ Faculty of Anesthesiology, Changhai Hospital, Naval Medical University, Shanghai 200433, China.; ^2^ Basic Medical University, Naval Medical University, Shanghai 200433, China.; ^3^ School of Anesthesiology, Naval Medical University, Shanghai 200433, China.; ^4^ Department of Clinic Genetics, Shanghai Changzheng Hospital, Naval Medical University, Shanghai 200433, China.; ^5^ Department of Critical Care Medicine, Naval Medical Center of PLA, Naval Medical University, Shanghai 200433, China.

## Abstract

Sepsis is a complex clinical syndrome marked by dysregulated immune responses, systemic inflammation, and subsequent organ dysfunction. Sepsis involves the interplay of multiple signaling pathways. Traditional sepsis treatment mainly depends on antibiotics and early directed therapy, with limited effectiveness. This article reviews major signaling pathways in sepsis, such as those related to nuclear factor κB (NF-κB), Janus kinase/signal transducer and activator of transcription (JAK/STAT), Toll-like receptors (TLRs), mitogen-activated protein kinase (MAPK), hypoxia-inducible factor 1α (HIF-1α), and nuclear factor-erythroid 2-related factor 2/Kelch-like ECH-associated protein 1 (Nrf2/Keap1). These molecules are pivotal in regulating immune activation, inflammation, and immune cell metabolism. Moreover, mitochondrial dysfunction and metabolic reprogramming substantially contribute to sepsis development, as they greatly affect energy production and immune cell function. Selectively inhibiting these pathways shows potential for effectively reducing hyperinflammation and preventing organ failure. We discussed how future research on these signaling pathways can translate into clinical applications and how personalized treatment strategies can handle the complexity and variability of sepsis. Given the dynamic nature of sepsis, treatment strategies should not solely rely on traditional single-target interventions. Instead, a dynamic and personalized multi-target modulatory approach is needed. While reducing side effects of single-target inhibition, inflammatory responses, immune balance, and metabolic disorders can be more precisely regulated. By precisely monitoring multiple sepsis-related signaling pathways and adjusting treatment regimens in real time, we aim to identify more effective intervention points in the complex dynamics of diseases, thus providing new hope for improving prognosis of septic patients.

## Introduction

The 2016 Sepsis 3.0 definition characterizes sepsis as life-threatening organ dysfunction that results from a dysregulated host response to infection [1]. The systemic impact of sepsis greatly influences immune function. On one hand, the overactivation of the immune response can result in an excessive inflammatory state. On the other hand, this heightened inflammatory response may trigger immunosuppression. This immunosuppression renders the host’s immune system dysfunctional and makes it susceptible to secondary infections. As a result, the clinical management of sepsis becomes more complicated [2]. Despite advancements in medical science, the treatment of sepsis continues to pose a substantial challenge. As initial interventions, standardized treatment protocols have been implemented, such as early goal-directed therapy and broad-spectrum antibiotic administration [3]. Nevertheless, these approaches have limitations due to the heterogeneity of sepsis presentations and variability in patient responses [4].

Despite numerous advancements in the field of sepsis research, notable deficiencies still remain. On one hand, multiple signaling pathways, including nuclear factor κB (NF-κB) and Janus kinase/signal transducer and activator of transcription (JAK/STAT), have been proven to be closely associated with sepsis [5]. However, the specific mechanisms of their interaction networks under different individual genetic backgrounds, preexisting disease conditions, and various stages of sepsis progression remain incompletely elucidated. On the other hand, existing targeted drugs for sepsis have shown varying efficacy and safety issues in clinical trials. For example, cytokine inhibitors have demonstrated a reduction in 1-month mortality and a decrease in the risk of severe adverse events. Conversely, Toll-like receptor 4 (TLR4) antagonists have been associated with an increased risk of adverse events [6]. Currently, there is a lack of effective methods to accurately predict the therapeutic response of sepsis patients to specific targeted drugs. Moreover, monitoring the immune function of sepsis patients is of crucial importance for sepsis management. Nevertheless, traditional indicators such as white blood cell count and C-reactive protein lack sensitivity and specificity. Recent studies have highlighted advanced biomarkers and techniques, such as programmed death 1/programmed death-ligand 1 (PD-1/PD-L1), human leukocyte antigen-DR (HLA-DR), specific cytokine profiles, as well as innovative technologies like flow cytometry, which offer more precise insights into the immune status of sepsis patients [7]. This indicates that the establishment of a precise and dynamic immune monitoring index system is essential for accurately assessing the immune function of sepsis patients and predicting disease prognosis.

In the diagnosis and treatment of sepsis, there are persistent clinical challenges and unmet medical needs. These have prompted the academic community to make the in-depth exploration of sepsis’s pathological mechanisms and related signaling pathways the core direction for formulating precise treatment strategies. This review focuses on the key signaling pathways related to the immune response and metabolic disorders during the progression of sepsis. It systematically integrates preclinical experimental data and clinical research evidence, as well as the latest research achievements in molecular and cellular biology.

The study mainly analyzes core signaling pathways, including NF-κB, JAK/STAT, and TLR. Moreover, it deeply interprets how these pathways regulate various pathological processes in sepsis. These processes cover immune regulation imbalance, uncontrolled inflammatory response, mitochondrial dysfunction, and metabolic reprogramming. We reveal the molecular targets of each signaling pathway and their upstream and downstream regulatory relationships. Then, by combining with the individual differences of patients in clinical practice, we explore potential treatment strategies based on these signaling pathways. This research not only provides a theoretical basis for the development of innovative targeted therapies for sepsis but also is committed to promoting the development of personalized medical strategies and achieving the precise customization of treatment plans. As our understanding of the complex cellular signaling network and metabolic remodeling mechanisms of sepsis continues to deepen, we expect to develop a new generation of therapeutic intervention methods. These methods can reduce the disease’s complexity and improve patient survival rates.

This review conducts an in-depth analysis of the signaling pathways with therapeutic potential, further highlighting the importance of targeted treatment strategies in tackling sepsis, a global health problem, and laying a solid theoretical foundation for improving the clinical prognosis of sepsis patients.

## Immune Regulation in Sepsis

Sepsis is a complex and life-threatening condition that involves a dysregulated immune response. The diversity of immune responses to sepsis is influenced by factors such as age (notably in young children and the elderly), infection type and location, genetics, and treatment strategies [8]. During sepsis, the activation of the immune system is triggered by a series of pattern recognition receptors (PRRs), which are receptors located on the surface of immune cells or within the cells. These receptors detect microbial-derived pathogen-associated molecular patterns (PAMPs), such as lipopolysaccharides from bacteria or viral nucleic acids, or endogenous damage-associated molecular patterns (DAMPs), released from damaged host cells, thereby initiating the host’s immune response. This recognition subsequently induces the release of cytokines and other inflammatory mediators or triggers cell death. Once detected, this recognition event sets off a chain reaction, leading to the release of cytokines and other inflammatory mediators or even triggering cell death. This initial immune activation is a fundamental defense mechanism of the body against invading pathogens [9].

However, sepsis is characterized by a severe dysregulation of the immune system. A key feature is the biphasic immune dysfunction, which consists of an initial, overwhelming inflammatory response followed by an immunosuppressive phase. This biphasic pattern is fundamental to the pathophysiology of sepsis [8]. The early hyperinflammatory phase results from an uncontrolled activation of leukocytes and endothelial cells, leading to a pro-inflammatory response. Concurrently, this triggers dysregulation in the production of oxygen and nitrogen radical species, cytokines, and the activation of the complement and coagulation systems [10]. As a consequence, although the activation of these mechanisms represents the body’s innate immune response to infection, an imbalance between the inflammatory and regulatory responses can result in excessive immunity causing tissue damage and accelerate the progression of sepsis [11].

Among different patients, the natural progression of sepsis shows variable adverse effects. Despite improvements in survival rates brought about by antibiotic treatments and supportive care, effectively managing disease progression remains a substantial challenge. This challenge is partly attributable to unresolved heterogeneity in patient immune imbalances [12]. Each patient’s immune system responds differently to sepsis, and understanding these individual differences is crucial for developing personalized treatment strategies.

### NF-κB signaling pathway

NF-κB signaling is a fundamental regulator of the immune response, bifurcating into 2 distinct pathways: the classical (canonical) and nonclassical (alternative) pathways [13]. Upon recognition of PAMPs by PRRs, such as TLRs, a complex signaling cascade is initiated. This cascade culminates in the activation and nuclear translocation of NF-κB. This translocation initiates the transcriptional expression of pro-inflammatory cytokines, chemokines, and adhesion molecules [14]. Upon stimulation, the IκB kinase (IKK) complex phosphorylates IκB, leading to its ubiquitination and proteasomal degradation. This process liberates NF-κB dimers, enabling their nuclear translocation and subsequent gene activation [15]. NF-κB dimers can translocate into the nucleus. Once in the nucleus, they bind to specific DNA target sites. This binding promotes the transcription of inflammatory cytokines such as tumor necrosis factor-α (TNF-α), interleukin-1β (IL-1β), and IL-6 [16].

**Fig. 1.**
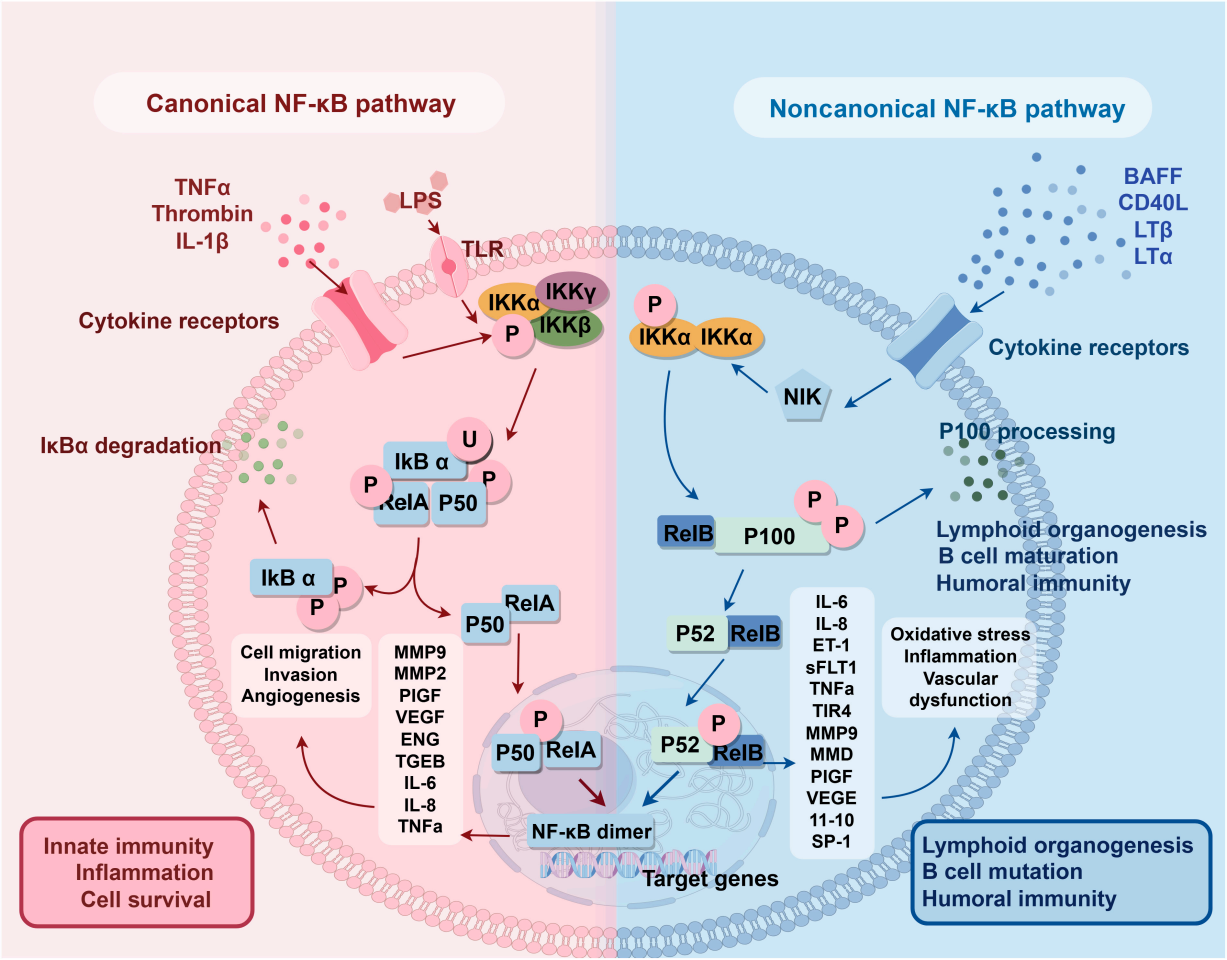
Molecular mechanisms of classical and nonclassical NF-κB pathways in sepsis pathogenesis. Sepsis is a life-threatening SIRS triggered by infection, often leading to MOF. The NF-κB signaling pathway is involved in this pathological process. Within the classical NF-κB pathway, sepsis-inducing agents, such as LPS, initiate a downstream signaling cascade through the activation of TLRs, culminating in the activation and nuclear translocation of NF-κB. Activated NF-κB enhances the transcription of inflammatory cytokines such as TNF-α and IL-1β, worsening systemic inflammation. NF-κB also regulates apoptosis and immune cell activation, crucial in sepsis development. The nonclassical NF-κB pathway involves unique activation mechanisms in specific cell types or stimuli, using different kinases, cofactors, and NF-κB subunit combinations. (By Figdraw.)

While the classical NF-κB pathway is a well-known mechanism for initiating immune responses, the nonclassical NF-κB signaling pathway also plays important roles in various physiological processes. It can be initiated by CD40 ligands, lymphotoxin β (LTβ), and bacterial lipopolysaccharide (LPS; also referred to as endotoxin), leading to the activation of NF-κB-inducing kinase (NIK) [17]. This pathway implies protein synthesis; thus, it operates with different kinetics than the classical one. It plays a crucial role in hematopoietic development, formation of secondary lymphoid organs, and maintenance of immune homeostasis [18]. A schematic representation of the mechanisms underlying both classical and nonclassical NF-κB pathways is provided in Fig. 1.

The interplay between pro-inflammatory cytokines and the NF-κB signaling pathway further complicates its role in the immune response. Pro-inflammatory cytokines sustain the NF-κB signaling pathway via an autocrine mechanism, a cascade that facilitates the recruitment of immune cells to the site of infection, thereby promoting pathogen elimination [19]. Nevertheless, in the context of sepsis, prolonged activation of NF-κB results in a persistent inflammatory response, which ultimately causes extensive tissue damage and organ failure [20].

Notably, NF-κB’s function extends beyond promoting inflammation. It also modulates the expression of genes related to anti-inflammatory pathways and tissue repair, highlighting its dual role in both propagating and resolving inflammation [21]. In sepsis, this delicate balance is often disrupted. Hyperactivation of NF-κB hinders inflammation resolution, causing continuous tissue damage [22]. In contrast, inadequate NF-κB activity in late stages of sepsis impairs tissue repair and leads to chronic immunosuppression [23]. As NF-κB plays a central role in sepsis, it represents a promising therapeutic target. By modulating its activity, we can enhance its protective effects while minimizing its harmful effects.

### JAK/STAT signaling pathway

There is a group of cytokines, such as IL-6, IL-11, and oncostatin M (OSM), that signal through the common receptor subunit glycoprotein 130 (gp130), activating downstream JAK/STAT pathways. Meanwhile, it also leads to the recruitment and phosphorylation of STAT family transcription factors. This group of mediators is sometimes referred to as IL-6-type cytokines. The JAK/STAT signaling pathway is also used by type 1 and type 2 cytokine receptors during sepsis. This pathway mediates the signaling of IL-6 and interferon-γ (IFN-γ) [24]. When cytokines bind to their specific receptors, JAK is activated. This activation causes the phosphorylation of STAT proteins, which then become activated. The activated STAT proteins translocate to the nucleus and regulate gene expression. This regulation controls immune cell differentiation, the production of inflammatory mediators, and the processes of cell survival and apoptosis [25]. The JAK/STAT signaling pathway is associated with the secretion of various cytokines and inflammatory mediators, such as IL-6, IL-10, inducible nitric oxide synthase (iNOS), and high mobility group protein B1 (HMGB1). This pathway plays a crucial role in modulating the immune response to sepsis [26].

The canonical JAK/STAT signaling pathway is typically initiated by extracellular signaling molecules, including cytokines, growth factors, and/or hormones, which bind to specific cell surface receptors. This triggers the phosphorylation and activation of adjacent JAK kinases [27]. Activated JAK kinase phosphorylates specific tyrosine residues on the receptor, which serve as docking sites for the recruitment of STAT proteins. This recruitment facilitates the phosphorylation of STAT proteins by JAK kinases [28]. Under specific circumstances, STAT proteins can be activated without relying on JAK. This activation can occur through 2 main alternative mechanisms. One is via growth factor receptors. The other is through nonreceptor tyrosine kinases, like the Src family kinases. These kinases can directly phosphorylate STAT proteins [29]. In later compensatory anti-inflammatory response syndrome (CARS), STAT3 inhibitors such as sunitinib or axitinib prevent the recruitment of myeloid-derived suppressor cells [30]. Inhibition of JAK3 and STAT3 has been shown to reduce the overexpression of endothelial adhesion molecules, thereby mitigating pathological conditions such as vascular paralysis, coagulation dysfunction, and multi-organ failure (MOF) associated with sepsis [31].

Beyond the primary components of the JAK/STAT signaling pathway, several proteins contribute to the STAT-dependent transcription process and facilitate the interaction of the JAK/STAT signaling pathway with other signaling pathways. These proteins are collectively referred to as regulators of JAK/STAT signaling [32]. In the context of the synergistic interaction between glucocorticoids and prolactin, activated STAT5 associates with GR to form a functional complex in which GR functions as a transcriptional coactivator for STAT5, thereby enhancing the transcriptional activity of STAT5-dependent genes [33]. At the same time, negative regulators also play a crucial role in modulating the JAK/STAT signaling pathway, thus collectively contributing to its stability and homeostasis [27]. The JAK/STAT signaling pathway has 3 main negative regulatory mechanisms. They are mediated by suppressor of cytokine signaling (SOCS) proteins, protein inhibitors of activated STAT (PIAS) proteins, and protein tyrosine phosphatases (PTPs) [34]. Among these, SOCS proteins play a crucial role in modulating the JAK/STAT pathway by attenuating its signaling activity [35]. Activated STAT proteins enter the nucleus to trigger SOCS transcription. SOCS proteins then deactivate JAK kinases by preventing STAT binding to receptors, by using their N-terminal kinase repressor domains, or by promoting ubiquitination and proteasomal degradation of JAKs or STATs, thereby negatively regulating the JAK/STAT signaling pathway [36]. By modulating the JAK/STAT signaling pathway, these inhibitors have the potential to attenuate hyperinflammation while maintaining the immune response.

**Fig. 2.**
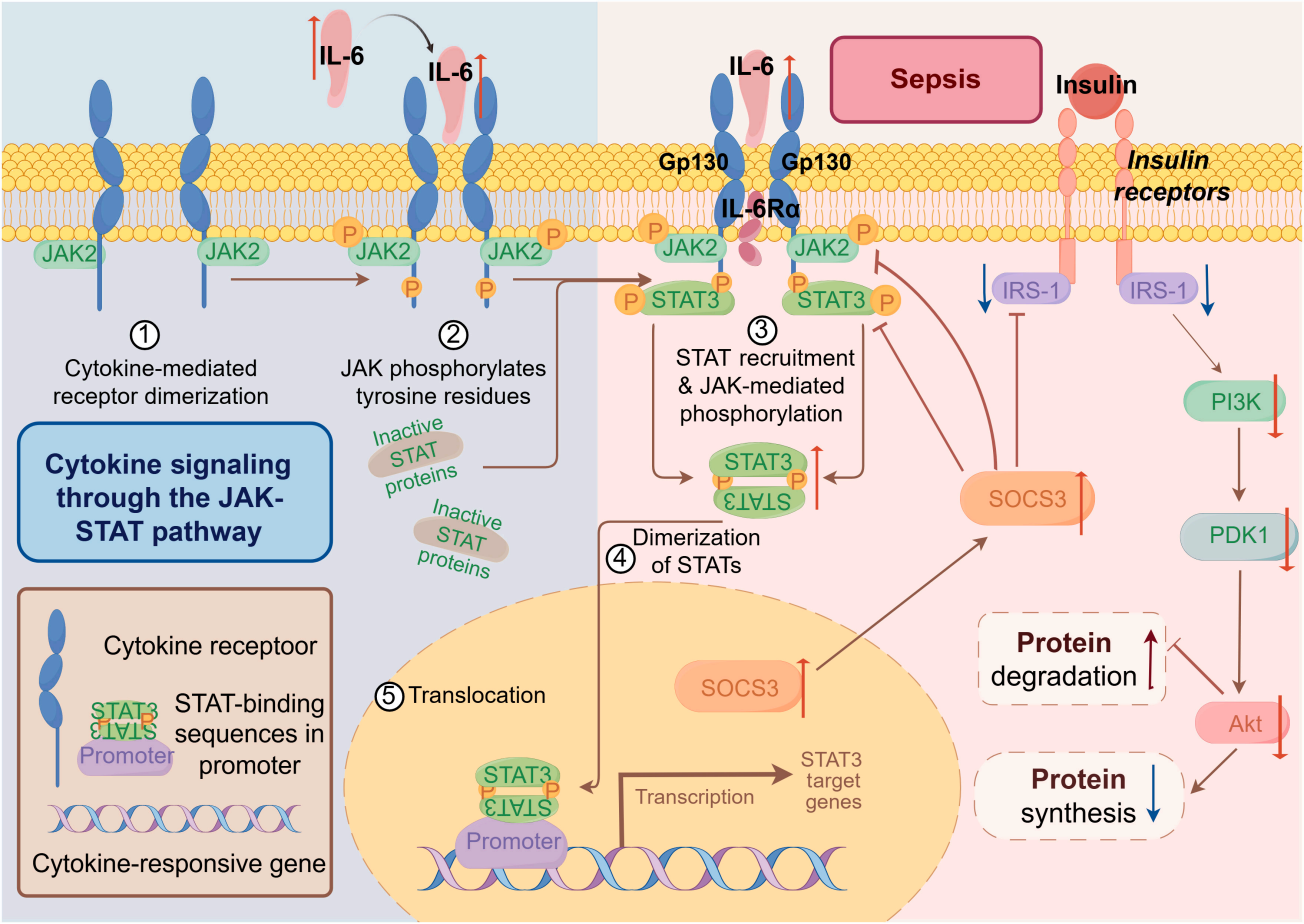
The transport and activation mechanisms of the JAK/STAT signaling pathway and its involvement in the pathophysiology of sepsis. Cytokines such as IFN-γ or IL-6 bind to cell surface receptors, activating JAK kinases, which phosphorylate STAT proteins. These phosphorylated STAT proteins dimerize and move to the nucleus and regulate genes related to inflammation, cell survival, and differentiation. Abnormal JAK/STAT activation can drive disease progression, impacting immune cell function and causing tissue damage and organ dysfunction. (By Figdraw.)

The JAK/STAT pathway modulates the fate and function of immune cells during sepsis. For instance, IL-6 activates STAT3 that initially provides anti-inflammatory protection in sepsis, but its prolonged activation may contribute to immune paralysis [37]. In contrast, IFN-γ activates STAT1 that triggers a pro-inflammatory response essential for pathogen clearance, but that if uncontrolled it may exacerbate tissue damage [38]. The balance between these opposing effects in JAK/STAT signaling is crucial for sepsis outcomes, making this pathway a promising target for therapy (Fig. 2).

While clinical applications of JAK/STAT inhibitors in sepsis have not yet been reported, their therapeutic use in inflammatory diseases and cancers is already well established. JAK inhibitors mainly inhibit the activation of downstream STAT proteins and block the signaling of inflammatory factors by targeting members of the JAK family [39]. The JAK/STAT pathway inhibitors have achieved remarkable results in the field of disease treatment, but they still face many challenges. In the future, it will be an important research direction to deeply explore the regulatory mechanism of JAK/STAT pathway and develop new inhibitors with high selectivity and low side effects, which is expected to bring new breakthroughs in the treatment of sepsis and other related diseases.

### TLR signaling pathway

TLRs are PRRs that function as primary responders within the innate immune system. They recognize conserved microbial structures referred to as PAMPs, thereby initiating an immune response [40]. In the pathological process of sepsis, TLR4 and TLR2, as key members of the pattern recognition receptor family, play important roles in mediating the host immune response by virtue of their unique structural characteristics and ligand-binding mechanisms.

TLR4, as the main receptor for recognizing Gram-negative bacteria, has a highly specific recognition ability for LPS, a component of the bacterial cell wall. By binding to TLR4, LPS triggers a series of cascade reactions, inducing host immune cells to release many inflammatory factors, thus driving the pathological progression of sepsis [41]. After activation, this receptor coordinately regulates the inflammatory response process through 2 signaling pathways, namely, the myeloid differentiation factor 88 (MyD88)-dependent and MyD88-independent pathways. The MyD88-dependent pathway mainly activates the NF-κB and mitogen-activated protein kinase (MAPK) signal transduction pathways, promoting the expression of pro-inflammatory cytokines such as TNF-α and IL-6. The MyD88-independent pathway, on the other hand, participates in immune regulation through the signal transduction mediated by the TIR domain-containing adapter-inducing interferon-β (TRIF) [42]. This dual-pathway regulation pattern endows TLR4 with complex and delicate regulatory functions in the inflammatory response and maintenance of immune homeostasis. In contrast to TLR4, TLR2 plays a crucial role in the recognition of Gram-positive bacteria. Its main recognition ligands include bacterial cell wall components such as lipopeptides and lipoteichoic acid [43]. After recognizing PAMPs, TLR2 recruits downstream adaptor proteins through the MyD88-dependent signaling pathway, activating the NF-κB and MAPK signaling pathways [44].

The activation of the TLR signaling pathway results in the swift production of pro-inflammatory cytokines and chemokines, which play a crucial role in controlling infections. Although TLR signaling is vital for pathogen recognition and immune activation, excessive or prolonged activation during sepsis has harmful effects [45]. Studies have shown that in TLR4-deficient mice, LPS-induced liver injury leads to several consequences. Liver enzyme levels decrease, and there are morphological changes in liver tissue. Additionally, oxidative stress occurs, and apoptosis is triggered, all of which differ from the wild-type controls. This suggests that sustained activation of TLR4 has a detrimental impact on organs such as the liver [46]. Thus, the inhibition of TLR signaling represents a potential therapeutic strategy to control hyperinflammation, address systemic autoimmune diseases, and manage systemic sepsis responses. Researchers have investigated various therapeutic approaches. These include using TLR antagonists, TLR-specific monoclonal antibodies (mAbs), and small molecules that can prevent TLR activation or block its downstream signaling pathways [47]. Notably, the synthetic lipid TLR4 antagonist Eritoran has demonstrated the ability in preclinical studies to attenuate LPS-induced inflammation and improve survival rates in sepsis models [48]. To achieve a more balanced immune response, researchers have developed more comprehensive and well-established methodologies. These mainly focus on 2 aspects: selectively targeting specific TLRs and modulating their signaling pathways, often in combination with other therapeutic strategies [49]. In summary, sepsis involves complex immune modulation through the NF-κB, JAK/STAT, and TLR pathways, emphasizing the need for targeted therapies. Exploring these mechanisms can uncover novel therapeutic targets to reduce sepsis-related damage and promote immune recovery.

### Microbiome metabolic pathways

The gut microbiome has been recognized as a crucial factor in the pathogenesis of sepsis, with its importance surpassing previous estimations. During sepsis, there is a disruption of both host metabolic processes and immune responses, wherein the gut microbiome plays an essential regulatory role [50]. The gastrointestinal system is postulated to be the “initiator” of sepsis-induced multi-organ dysfunction syndrome (MODS) and is instrumental in triggering and sustaining various mechanisms of systemic inflammatory response. Clinically, intestinal dysbiosis is frequently observed in patients with sepsis characterized by an imbalance in the gut microbiota, which correlates with adverse outcomes [51]. Specifically, SCFAs such as acetate, propionate, and butyrate have been shown to play a role in the signaling processes between bacterial communities and the immune system [52]. In addition to SCFAs, multiple metabolites of gut microbiota such as amino acids and their metabolic derivatives play critical roles in the occurrence and development of sepsis. For example, some microbial metabolites can up-regulate the expression of the iron transporter transferrin, and transferrin can exert immunosuppressive functions through interaction with CD14, specifically by inhibiting PRR signaling, thereby inducing host immune tolerance [53]. Sepsis patients often exhibit disorders in amino acid metabolism, which may lead to reduced immune function and intestinal barrier damage. For example, L-valine is an important amino acid, and the decrease in its metabolite levels is closely associated with poor prognosis in sepsis patients. This indicates that amino acid metabolites can serve as potential biomarkers for sepsis, thereby providing new insights for clinical diagnosis and treatment [54]. Indole-3-propionic acid (IPA), a gut microbiota metabolite mainly derived from tryptophan, has notable anti-inflammatory effects. IPA can up-regulate the expression of anti-inflammatory factors and inhibit the secretion of pro-inflammatory factors, thus exerting a protective role in inflammatory conditions such as sepsis [55]. In septic mice, IPA treatment reduced serum levels of inflammatory mediators, improved survival rate, altered the composition of gut microbiota by enhancing the abundance of beneficial bacteria such as Bifidobacterium, and reduced the number of potential pathogenic bacteria in the intestine. These effects help enhance the integrity of the intestinal barrier, alleviate intestinal damage, and thereby improve the survival rate of mice [56]. Research on gut microbiota metabolites in sepsis provides new insights, highlighting their crucial role in maintaining intestinal barrier function and the effectiveness of immune responses.

Metabolomics, defined as the analysis of metabolites within biological systems, has emerged as a powerful method for uncovering metabolic alterations associated with sepsis [57]. For instance, reduced concentrations of SCFAs produced by gut microbiota are correlated with compromised intestinal barrier function and increased systemic inflammation in septic conditions [58]. Recent preclinical studies have shown that fecal microbiota transplantation can restore the original SCFA-producing microbial community in mice with sepsis, restoring the immune function and controlling over proliferating pathogens [59]. Interventions like enteral fiber-rich nutrition can restore a healthy microbiota. This restoration has the potential to modulate metabolic processes and enhance the immune response in sepsis [60]. These therapies may facilitate the re-establishment of beneficial metabolite production and promote a balanced immune response, thereby enhancing patient outcomes.

Future research directions may include multi-omics integration analysis to decode the gut microbiota metabolite network. Through multi-omics integration analysis, the complex interactions between gut microbiota and the host can be revealed. When performing multi-omics integration analysis, by collecting different types of omics data, including genomics, transcriptomics, metabolomics, etc., we can more comprehensively understand the specific roles of microorganisms in host metabolism [61]. With the deepening of research, it is expected that more refined integration analysis will discover new biomarkers and therapeutic targets to improve the prevention and treatment of microbiota-related diseases. In addition, the development of personalized microbiota metabolite treatment strategies has become an important research direction in the field of microbiome research in recent years. This strategy not only focuses on the composition of the microbiota but also emphasizes the role of its metabolites in disease occurrence and progression. In personalized treatment, analyzing the composition of a patient’s gut microbiota and its metabolites can enable the formulation of more precise treatment strategies for different patients [62]. The data from multi-omics integration analysis will provide stronger support for the development of personalized microbiota metabolite treatment strategies. Furthermore, efforts should be dedicated to revealing the causal relationships between gut microbiota and sepsis, elucidating their mechanisms and metabolites, and developing effective interventions. Ultimately, a deep understanding of the interactions between gut microbiota metabolites and the host is expected to provide new directions for improving the prognosis and survival rate of sepsis patients. With the continuous advancement of research, we anticipate that the relationship between gut microbiota and sepsis will bring revolutionary breakthroughs to clinical treatment, improving patients’ quality of life and life expectancy.

### Other sepsis immune-related signaling pathways

In addition to the aforementioned signaling pathways that play a critical role in the immune response of sepsis patients, the complement system pathways and the NOD-like receptor protein 3 (NLRP3) inflammasome signaling pathways contribute to immune regulation in sepsis [63]. In mammals, the complement system consists of 3 primary activation pathways: the classical pathway, the alternative pathway, and the lectin pathway [64]. The classical pathway gets activated when antibodies bind to pathogen surface antigens. This binding triggers a cascade reaction. First, it involves the activation of C1q, followed by C4, C2, and C3. Ultimately, this cascade leads to the formation of a membrane attack complex (MAC). The MAC is responsible for lysing, or destroying, the pathogen [65]. In contrast, the alternative pathway activates C3 without antibodies by directly recognizing pathogen surface structures and follows a similar process to form MAC [66]. Unlike the classical and alternative pathways, the lectin pathway is triggered by the pathogen’s surface glycostructure. Specifically, it gets activated when mannose-binding lectin (MBL) or other lectins bind to the pathogen, thereby activating the complement system. This activation not only helps clear pathogens quickly but also generates inflammatory mediators such as C3a and C5a. These mediators play a crucial role in attracting immune cells and enhancing the overall inflammatory response [67].

**Fig. 3.**
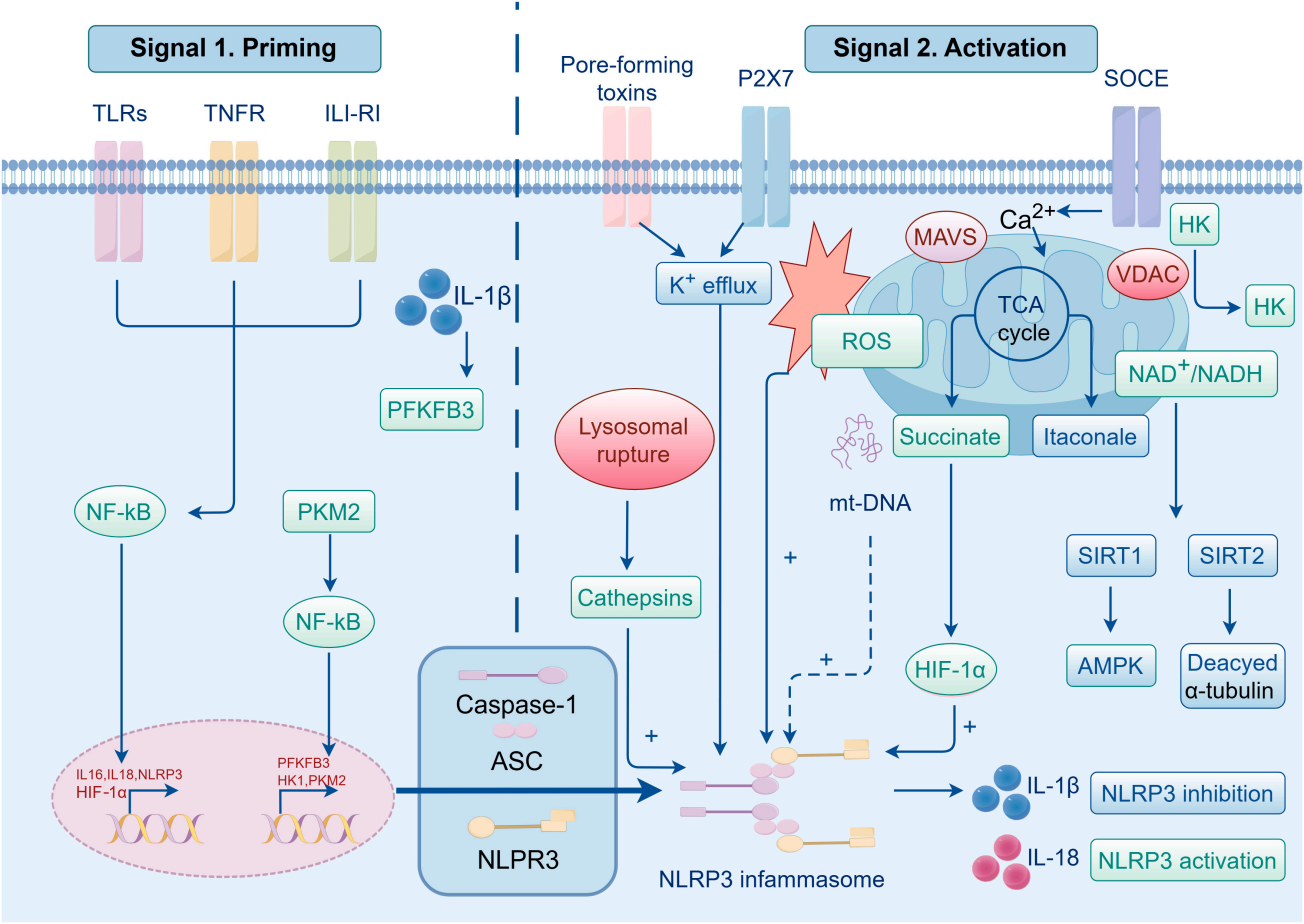
Activation mechanism of the NLRP3 inflammasome in sepsis: A dual-signaling model. In the dual-signaling model, NLRP3 inflammasome activation involves 2 steps: First, NLRP3 expression is up-regulated by PAMPs or DAMPs; second, NLRP3 is activated through intracellular events such as potassium efflux and lysosomal rupture. Activated NLRP3 binds to apoptosis-associated speck-like proteins containing a CARD (ASC) and procaspase-1 to form the inflammasome complex. By cleaving procaspase-1 into active caspase-1, this complex promotes the maturation and release of inflammatory factors such as IL-1 and IL-18. (By Figdraw.)

The NLRP3 inflammasome is a protein complex that regulates caspase-1 activation and the release of pro-inflammatory cytokines such as IL-1β and IL-18 [68]. Besides, it has been found to initiate apoptosis through nonconventional pathways. In terms of its activation mechanism, when the NLRP3 inflammasome is activated by LPS, it leads to the activation of caspase-11. This, in turn, triggers a process known as pyroptosis, a form of programmed cell death that relies on the activation of inflammasomes [69]. NLRP3 inflammasome contributes to sepsis development, and its inhibition can lessen its severity [70]. The dual-signal activation mechanism of NLRP3 in sepsis is illustrated in Fig. 3.

We outline in Table 1 the additional immune-related signaling pathways that contribute to the inflammatory response and immunosuppression observed in sepsis.

**Table 1.** Immune signaling pathways’ role in organ damage in sepsis

Signaling pathways	Organs	Mechanism	Effect of the intervention	References
High mobility group box 1 (HMGB1)	Lung	HMGB1 has the capacity to directly interact with alveolar epithelial cells, resulting in cellular damage and initiation of an inflammatory response.	During acute lung injury (ALI), HMGB1 facilitates the polarization of macrophages toward the M1 phenotype and exacerbates ALI via the NF-κB signaling pathway mediated by TLR2 and TLR4.	[163]
	Brain	HMGB1 exacerbates the occurrence and development of delayed neuroinflammation following ischemia by inducing the production of cytokines and promoting intercellular communication.	HMGB1 leads to neuronal death, microglial activation, and exacerbated brain injury. Targeting HMGB1 may represent a novel therapeutic strategy for sepsis-associated brain damage.	[164]
Notch	Heart	In the context of sepsis, the Notch signaling pathway influences endothelial cell fate by modulating angiogenesis, endothelial cell apoptosis, and vascular permeability.	The ZFAS1/Notch signaling pathway is subject to regulation by the transcription factor Sp1, which ameliorates myocardial injury associated with sepsis.	[165]
	Lung	In sepsis, dysregulated activation of the Notch signaling pathway contributes to the regulation of alveolar epithelial cell proliferation, differentiation, and apoptosis, potentially leading to alveolar epithelial injury and exacerbating lung damage.	γ-Secretase inhibitors (GSIs) suppress Notch signaling, reducing alveolar epithelial cell apoptosis, alleviating inflammatory cell infiltration, and mitigating lung injury in septic mice.	[166]
Transforming growth factor β (TGF-β)	Kidney	TGF-β promotes fibroblast proliferation and extracellular matrix synthesis, which may lead to renal fibrosis and impair renal function.	In a sepsis-induced mouse model of acute kidney injury, the transcription factor USF2 activates the TGF-β signaling pathway, thereby promoting pyroptosis and organ damage. Inhibition of the TGF-β signaling pathway alleviates fibrosis and protects renal function.	[167]
Wnt/β-catenin	Kidney	Upon organ injury, the Wnt/β-catenin pathway is activated, which induces renal epithelial cell apoptosis and inflammatory responses during sepsis and exerts effects on cell tissue regeneration.	Up-regulated expression of microRNA-23a-3p inhibits the Wnt/β-catenin signaling pathway, thereby suppressing cell apoptosis, reducing the release of proinflammatory cytokines, and alleviating sepsis-induced acute kidney injury.	[168]
RAS/RAF/MEK/extracellular signal-regulated kinases (ERKs)	Lung	The RAS/RAF/MEK/ERK signaling cascade is a key component of the MAPK pathway. Inhibition of the ERK/NOX2 signaling pathway alleviates sepsis-induced lung injury.	Small-molecule inhibitors targeting the RAS/RAF/MEK/ERK signaling pathway hold potential therapeutic value in sepsis treatment.	[169]
PI3K/protein kinase B (Akt)	Lung	The PI3K/Akt signaling pathway may regulate the immune function of neutrophils during sepsis by modulating processes such as glycolysis in these cells.	Shenfu (SF) injection alleviates inflammatory responses, oxidative stress injury, and apoptosis by inhibiting the PI3K/Akt signaling pathway, thereby contributing to the improvement of acute respiratory distress syndrome in septic patients.	[170]
	Heart	Inhibition of the PI3K/Akt signaling pathway not only induces apoptosis in cardiomyocytes but also exacerbates inflammatory responses, causing damage to the structure and function of the heart.	Quercetin inhibits LPS-induced apoptosis, inflammatory responses, and ferroptosis. Knockdown of arachidonic acid 5-lipoxygenase (ALOX5) synergistically suppresses these pathological processes, while ALOX5 overexpression counteracts the protective effect of quercetin against LPS-induced myocardial injury.	[171]
DNA-PKcs/cofilin2	Heart	DNA-PKcs phosphorylates cofilin2 at Thr^25^, promoting F-actin depolymerization, disrupting endothelial barrier integrity, leading to myocardial microcirculatory disorder.	Inhibition of DNA-PKcs or expression of phospho-defective cofilin2 ameliorates endothelial dysfunction and improves heart function in endotoxemic mice.	[172]

The aforementioned immune-related signaling pathways play a crucial role in immune modulation during sepsis, and their aberrant activation often triggers inflammatory reactions. The inflammatory response in sepsis is highly complex, involving interactions among various cells and molecules, closely associated with immune regulation. Below, we will delve into the inflammatory response in sepsis and its related signaling pathways.

## Inflammatory Response in Sepsis

Sepsis-associated inflammatory response is mainly mediated by leukocytes. This response also involves a complex interaction among cytokines, ROS, endothelial cells, the complement system, and the coagulation system [71]. Neutrophils play a role in the recognition and elimination of invading pathogens and in maintaining immune homeostasis [72]. An expanding corpus of evidence suggests that targeting neutrophils holds promise as a therapeutic approach in sepsis treatment [73]. For instance, activated granulocytes released from the bone marrow form NETs to ensnare pathogenic microorganisms, a process that amplifies the inflammatory response in sepsis contributing to tissue damage [74]. Beyond the role of neutrophils, the inflammatory response observed in sepsis is characterized by concurrent neuroendocrine alterations, activation of the complement and coagulation pathways, and modifications in lipid mediators, all of which synergistically contribute to the amplification of the inflammatory state [75]. This response is a complex process that plays a critical role in defending against infection and preventing further tissue damage. In this section, we will explore signaling pathways involved in the inflammatory response during sepsis, with a particular focus on the MAPK, hypoxia-inducible factor 1α (HIF-1α), and nuclear factor-erythroid 2-related factor 2/Kelch-like ECH-associated protein 1 (Nrf2/Keap1) pathways. A deeper understanding of these pathways is essential for identifying potential therapeutic targets that could modulate the inflammatory process and improve clinical outcomes.

### MAPK signaling pathway

MAPKs, as highly conserved serine/threonine kinases expressed in nucleated cells and platelets, have downstream apoptosis signal-regulating kinase 1 (ASK1) belonging to the mitogen-activated protein kinase kinase kinase (MAP3K) family, which can activate downstream mitogen-activated protein kinase kinase (MAP2K) upon stress stimulation [76]. MAP2Ks sequentially phosphorylate downstream MAPKs, including extracellular signal–regulated kinases (ERKs), c-Jun N-terminal kinases (JNKs), and p38 MAPKs [77]. Each of these signaling pathways plays a distinct role in modulating immune cell activation, cytokine production, and apoptosis in the context of sepsis [78]. When the body detects PAMPs and DAMPs, these MAPK pathways are activated. This activation triggers a series of inflammatory signaling events. Eventually, it leads to an excessive release of cytokines, a phenomenon commonly known as the “cytokine storm”. This storm is frequently observed in sepsis patients [79]. The TLR4/JNK signaling pathway modulates the production of pro-inflammatory cytokines and myocardial dysfunction induced by LPS. In animal experiments using mouse models of sepsis, researchers applied the inhibitor TAK-242 to block TLR4 activation. As a result, JNK activation was reduced, the plasma concentration of TNF-α protein was decreased, and histological damage to the myocardium was lessened. These effects together helped maintain cardiac function [80].

Considering the function of MAPK pathways in regulating inflammatory responses during sepsis, the targeted inhibition of these pathways emerges as a promising therapeutic strategy [81]. Inhibition of ASK1, whether through gene knockout, pharmacological intervention, or suppression of upstream factors, leads to a reduction in p38 MAPK phosphorylation and TLR-mediated cytokine production. Thus, ASK1 is a crucial signaling component in innate immunity [82]. ASK1 plays a critical role in the regulation of immune-mediated thrombocytopenia, thrombosis, and systemic shock. Consequently, the inhibition of ASK1 serves as a promising therapeutic target for the treatment of immune complex (IC)-induced shock and other immune-mediated thrombotic disorders [83].

### HIF-1α signaling pathway

**Fig. 4.**
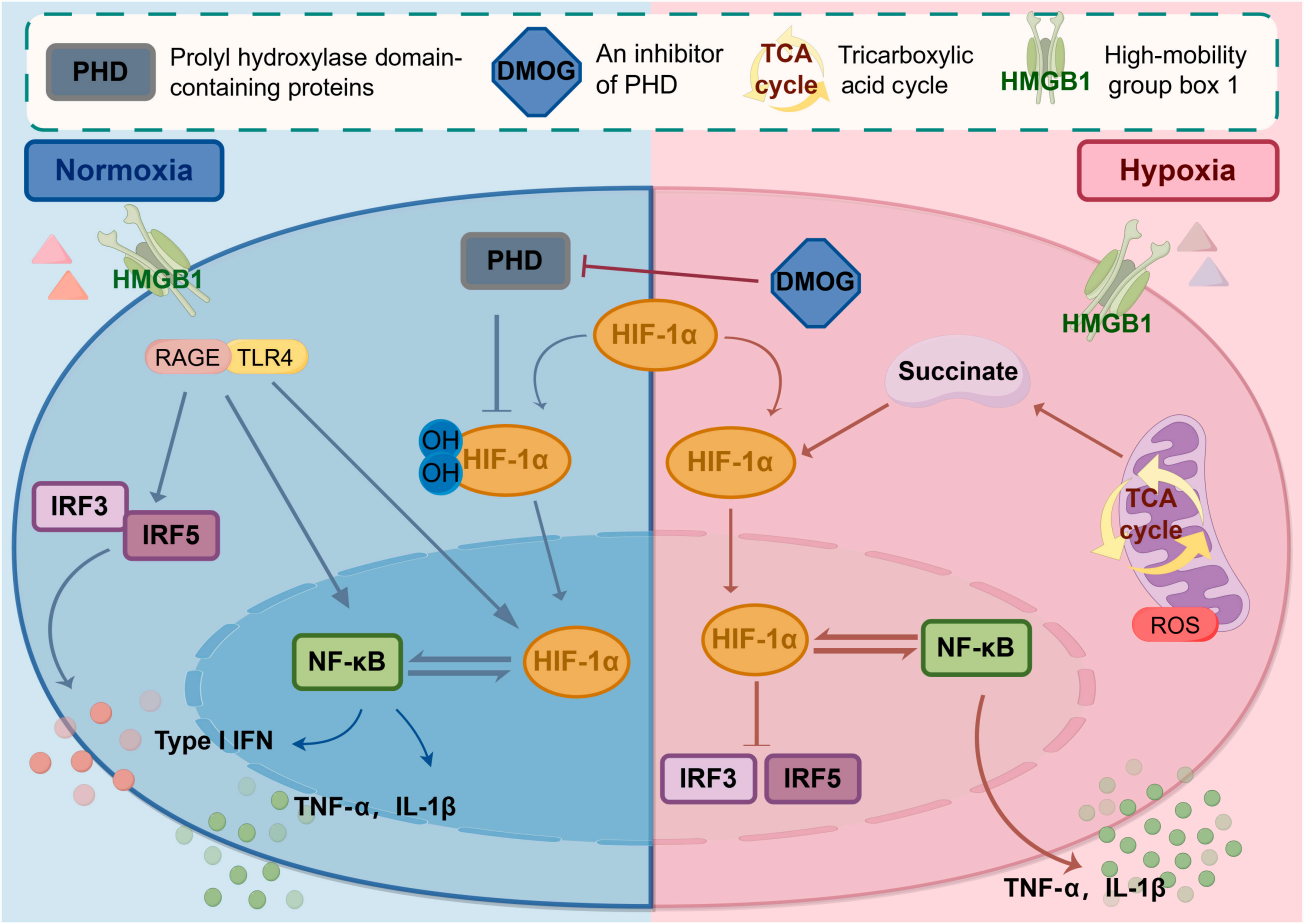
The mechanism of action of HIF-1α in normoxia versus hypoxia inflammatory milieu characteristic of sepsis. In hypoxic environments, both the stability and activity of HIF-1α are increased, activating downstream signaling pathways that influence the regulation of inflammation, immune responses, and organ dysfunction. HIF-1α directly modulates the expression of inflammatory genes and interacts with the NF-κB signaling pathway. NF-κB, a pivotal inflammatory regulator, similarly plays a crucial role in sepsis. During the onset and progression of sepsis, the activation of NF-κB results in the excessive production of inflammatory mediators. (By Figdraw.)

Multiple pathophysiological mechanisms contribute to the development of MODS in septic patients. Vascular endothelial damage, cellular dysfunction, and activation of the coagulation system are among the key culprits. These factors interact in complex ways. Ultimately, they cause organ and tissue hypoperfusion, intravascular microthrombosis, and the failure of both macrocirculation and microcirculation [84]. Tissue hypoperfusion caused by sepsis leads to tissue hypoxia and impairs cellular oxygen utilization. This process generates free radicals through mechanisms similar to those seen in oxidative stress during ischemia–reperfusion injury [85]. HIF-1α is a component of the heterodimeric protein complex HIF-1. It regulates cellular responses to physiological hypoxia and infection, and is often associated with acute hypoxic conditions [86]. Its primary role is the activation of genes associated with the glycolytic pathway, which decreases cellular oxygen consumption and mitigates the production of ROS. The activation of HIF-1α facilitates the adaptation of immune cell metabolism to anaerobic conditions [87]. The schematic illustration of the role of HIF-1α in both normal and hypoxic inflammation is presented in Fig. 4.

Sepsis is characterized by a dysregulated inflammatory response to infection. Blood monocytes, as the host’s initial line of defense, play a crucial role in this process. These cells can effectively detect invading pathogens and rapidly trigger an immune inflammatory response to effectively combat the infection [88]. Under hypoxic conditions, although monocytes can still produce pro-inflammatory cytokines through the action of HMGB1, the generation of IFN1s is suppressed. This suppression can be attributed to hypoxia-induced HIF-1α, which acts as a direct transcriptional repressor of IRF5 and IRF3 [89]. As a result, HIF-1α modifies monocyte metabolism, promoting the release of pro-inflammatory cytokines while keeping IFN levels stable. Evidence indicates that hypoxia and HIF-1α affect bacterial phagocytosis. In group B streptococcus infection, when HIF-1α is deleted in monocytes, the intracellular killing and lysing abilities of bacteria decrease. However, the rate of phagocytic uptake is mostly unchanged [90]. HIF-1α both promotes the production of pro-inflammatory cytokines and supports cell survival. This dual function makes regulating HIF-1α in sepsis a complex and challenging task with 2 distinct aspects [91]. Increased expression of HIF-1α increases inflammatory response, potentially resulting in tissue damage and organ dysfunction, particularly when hypoxic conditions extend beyond the acute phase [92]. Targeted therapies aimed at HIF-1α represent a promising strategy for mitigating organ damage in sepsis. HIF-1α inhibitors, in particular, have the potential to attenuate excessive inflammatory responses while preserving essential cellular functions [93]. Future research should investigate the impact of HIF-1α regulation on both inflammation and the metabolic adaptation of immune cells in the context of sepsis.

### Nrf2/Keap1 signaling pathway

The transcription factor Nrf2 belongs to the leucine zipper transcription factor family within the human Cap’n’Collar (CNC) domain [94]. Under physiological conditions, Nrf2 is sequestered in the cytoplasm by its inhibitor, Keap1 [95]. In response to oxidative stress, Nrf2 is released and translocated to the nucleus, where it initiates transcription of genes associated with antioxidant defense and detoxification [96]. The Nrf2-Keap1 axis is essential for disease prevention due to its role in regulating antioxidant, detoxifying, and anti-inflammatory responses [97]. This pathway is crucial for managing oxidative stress, which is elevated during sepsis [98].

Currently, pharmacological agents aimed at the Keap1-Nrf2 signaling pathway work via 2 mechanisms. Firstly, they enhance the accumulation and nuclear translocation of Nrf2. They do this by disrupting the binding between Nrf2 and Keap1 through oxidation, alkylation modification, or competitive binding. As a result, the body’s antioxidative stress response is augmented [99]. Secondly, these agents help Keap1 to dissociate from Nrf2. They achieve this by increasing Nrf2 phosphorylation. This increase in phosphorylation also promotes the accumulation of Nrf2 in the nucleus [100]. Nrf2 regulates both antioxidant and inflammatory pathways. In vitro studies show that Keap1-deficient macrophages have a reduced inflammatory response to TLR agonists, whereas Nrf2 deficiency increases the inflammatory response to these agonists [101]. The Nrf2 pathway is considered crucial for cancer progression, metastasis, and resistance to radiotherapy. Inhibiting Nrf2 can potentially reduce the survival and proliferation of cancer cells. At the same time, it can enhance the tumor’s sensitivity to chemotherapy and radiotherapy. This is accomplished by modulating immune responses, increasing DNA damage, and reducing antioxidant defenses [102]. Additional clinical data and in vitro and in vivo experiments are required to enhance our understanding of the therapeutic window for Nrf2 activation and its interactions with other signaling pathways involved in sepsis. A deeper understanding of the intricate signaling pathways implicated in this response, such as MAPK, HIF-1α, and Nrf2/Keap1, is essential for the advancement of targeted therapeutic strategies aimed at modulating inflammation and safeguarding organ function.

### Additional signaling pathways associated with inflammation in sepsis

In addition to the signaling pathways implicated in immune regulation during sepsis, numerous other pathways are associated with the inflammatory response in this condition, extending beyond the aforementioned ones. These include the cGAS-STING signaling pathway, the inflammatory factor/cytokine pathway, and the PI3K/Akt signaling pathway, among others. The cyclic guanosine monophosphate (GMP)–adenosine monophosphate (AMP) synthase (cGAS) and stimulator of interferon genes (STING) pathway represents a novel DNA-sensing mechanism that has been identified in recent years [103]. Activation of STING triggers the production of IFNs and various inflammatory mediators, with excessive activation potentially resulting in cardiovascular complications, including myocardial infarction, heart failure, and myocarditis [104].

In the context of sepsis, oxidative stress is exacerbated, and its products can inhibit PI3K activity, diminish Akt phosphorylation levels, and impair the PI3K/Akt signaling pathway, ultimately leading to a reduction in the inflammatory response [105]. Furthermore, the PI3K/Akt signaling pathway regulates the expression of proteins associated with apoptosis, a process commonly observed in sepsis [106]. Notably, heme oxygenase-1 (HO-1), known for its anti-inflammatory properties, has been shown to inhibit the PI3K/Akt signaling pathway during sepsis-induced lung injury in murine models. This inhibition modulates autophagy and reduces inflammation, resulting in improved lung function and increased survival of these mice [107]. Numerous additional signaling pathways are implicated in the regulation of the inflammatory response and contribute to the pathogenesis of systemic organ injury in sepsis (Table 2).

**Table 2.** The role of inflammatory signaling pathways in sepsis-mediated organ damage

Signaling pathways	Organs	Mechanism	Effect of the intervention	References
HMGB1-RAGE	Lung	In the context of LPS-induced ALI, HMGB1 contributes to pathogenesis by activating the AIM2 inflammasome within macrophages and promoting M1 macrophage polarization through the TLR2, TLR4, and RAGE/NF-κB signaling pathways.	Crataegin reduces histopathological damage and mortality in septic mice by inhibiting HMGB1 production. Additionally, its inhibitory effects on RAGE and TLR2/4 block the HMGB1-RAGE and HMGB1-TLR signaling pathways.	[173]
	Liver	Sepsis induces hepatocyte injury and promotes the massive release of HMGB1. Activation of the HMGB1-RAGE signaling pathway exacerbates hepatocyte pyroptosis and autophagy, thereby leading to further deterioration of liver function.	Anti-HMGB1 antibodies or RAGE inhibitors alleviate hepatocyte injury and inflammatory responses. Resveratrol attenuates hepatocyte inflammatory responses by up-regulating SIRT1 expression to inhibit HMGB1 release and nucleocytoplasmic translocation.	[174]
Wnt/β-catenin	Kidney	In acute kidney injury, transient activation of the Wnt/β-catenin pathway promotes tissue regeneration and repair. Conversely, prolonged activation leads to renal tubular epithelial cell damage, thereby inducing renal fibrosis, inflammation, and kidney injury.	The Wnt/β-catenin signaling pathway promotes tissue regeneration and renal function recovery in AKI. However, its prolonged activation in CKD exacerbates tubular injury, fibrosis, and inflammation. Interactions between Wnt/β-catenin and pathways such as Notch provide new directions for combined interventions in kidney diseases.	[175]
	Heart	Inflammatory responses associated with sepsis activate the Wnt/β-catenin signaling pathway, triggering the activation of downstream target genes including NF-κB. This process promotes myocardial cell hypertrophy, apoptosis, and dysfunction, ultimately leading to impaired cardiac function.	In a mouse model of cardiac hypertrophy and heart failure induced by transverse aortic constriction, the Wnt/β-catenin signaling pathway is activated in both cardiac and renal tissues. Inhibition of this pathway has been shown to improve tissue damage in both organs.	[176]
Autophagy	Heart	In the early stage of sepsis, autophagy exerts a protective effect by clearing damaged components, while excessive autophagy in the late stage induces apoptosis and tissue damage. The PI3K/Akt signaling pathway can enhance autophagic activity, inhibit apoptosis, and maintain myocardial function.	Drugs such as dapagliflozin can improve sepsis-induced cardiac injury by regulating signaling pathways including autophagy and PI3K/Akt.	[177]
	Lung	In sepsis, reduced autophagy impairs the clearance of damaged cellular components and inflammatory mediators, thereby leading to exacerbated inflammatory responses and lung tissue damage.	Enhancing lysosomal acidification and restoring autophagy can mitigate lung injury and improve the survival rate of septic mice. Autophagy inducers like rapamycin can reduce cellular damage, inflammatory responses, and sepsis-associated lung injury.	[178]
	Kidney	Autophagy inhibits the activation of inflammasomes by promoting the degradation of damaged mitochondria and lysosomes, thereby reducing the release of DAMPs.	Activation of autophagy can alleviate sepsis-induced renal injury. Therefore, targeting autophagy may represent a promising therapeutic strategy for managing sepsis-associated renal injury.	[179]
Ang-II	Heart	In sepsis, angiotensin II activates the NF-κB pathway via AT1 receptors, with its mediated inflammation and oxidative stress exacerbating myocardial injury. Meanwhile, vasoconstriction induces myocardial remodeling and cardiac dysfunction.	Angiotensin-converting enzyme inhibitors (ACE inhibitors) and angiotensin receptor blockers (ARBs) can reduce inflammation, inhibit fibrosis, maintain myocardial function, and enhance cardiac contractility.	[180]
	Kidney	Sepsis can activate the renin–angiotensin–aldosterone system (RAAS), promoting increased production of angiotensin II (Ang-II), exacerbating vasoconstriction and renal hypoperfusion, and ultimately leading to renal ischemic injury.	The use of RAAS antagonists, particularly ACE inhibitors and ARBs, can reduce renal tissue injury and the incidence of sepsis-induced acute kidney injury.	[181]
	Liver	Angiotensin II exacerbates septic hepatocyte injury and dysfunction by promoting inflammation, oxidative stress, and apoptosis. It also causes hepatic vasoconstriction, worsening ischemia and hypoxia to further impair liver function. Additionally, angiotensin II inhibits bile secretion, leading to cholestasis and additional liver damage.	In patients with decompensated cirrhosis when mean arterial pressure cannot be maintained with conventional vasopressors, angiotensin II may serve as a potential adjunctive resuscitative agent.	[182]
NLRP3 inflammasomes	Heart	Activation of the NLRP3 inflammasome promotes the release of pro-inflammatory cytokines, leading to myocardial cell injury and dysfunction. It may also activate apoptosis-related proteins such as caspase-3 to induce myocardial cell apoptosis.	Chrysophanol reduces inflammation and myocardial pyroptosis by inhibiting the NLRP3 inflammasome in septic mice.	[183]

The inflammatory response in sepsis exerts extensive effects on the body through various signaling pathways, and the persistence of inflammation can further lead to cellular dysfunction. Mitochondria, as crucial organelles within cells, are highly susceptible to impairment during the pathogenesis of sepsis. We will now explore the relevant mechanisms underlying mitochondrial dysfunction in sepsis.

## Mitochondrial Dysfunction in Sepsis

Mitochondria are the primary energy generators within cells, and they are key regulators of cellular apoptosis and immune responses [108]. Mitochondrial dysfunction constitutes a critical component of the pathophysiology of sepsis, contributing to the shift from adaptive immune responses to pathological inflammation and subsequent organ failure [109]. In the context of sepsis, mitochondrial function is markedly compromised due to systemic inflammation, hypoxia, and oxidative stress, leading to diminished biosynthetic activity, reduced ATP production, cellular energy depletion, increased ROS generation, and the initiation of apoptosis [110]. Assessing mitochondrial metabolism through cellular respiration provides a valuable means to monitor treatment responses in patients with acute conditions, such as sepsis, and to identify individuals at risk of progressing to organ failure [111].

### Impaired mitochondrial bioenergetics

Sepsis-induced mitochondrial dysfunction often leads to impaired bioenergetics. This impairment occurs because excessive production of ROS and inflammatory mediators damages mitochondrial DNA, proteins, and membranes [112]. When mitochondrial bioenergetics are impaired, the efficiency of the OXPHOS process decreases. As a direct consequence, ATP production decreases, causing an energy deficit. This energy deficit has a more severe impact on tissues with high metabolic demands [113]. Furthermore, the transition from aerobic respiration to anaerobic glycolysis as an alternative energy source results in accumulation of lactate. Elevated lactate levels are indicative of sepsis in patients and suggest cellular hypoxia and metabolic acidosis [114]. The disruption of mitochondrial bioenergetics plays a crucial role in the development of sepsis. It directly causes mitochondrial dysfunction and reduces oxidative ATP production. These consequences can ultimately lead to organ failure and increase the risk of mortality [115].

### Role of mitochondrial ROS in sepsis

Mitochondrial metabolism is a source of ROS. Optimal levels of ROS are essential for cellular signaling and host defense mechanisms. During sepsis, the dysregulation of mitochondrial ROS production results in the malfunctioning of the intracellular antioxidant system. This dysfunction contributes to oxidative damage to cellular components and exacerbates the inflammatory response [116]. Excessive ROS production induces inflammasome activation, further amplifying the inflammatory cascade [117]. Consequently, targeting mitochondrial ROS using antioxidants or ROS scavengers presents a promising therapeutic approach to mitigate oxidative stress and inflammation associated with sepsis [118]. Systematic reviews and meta-analyses have synthesized evidence from multiple studies. The results of these comprehensive analyses suggest that antioxidant therapy may be effective in reducing the short-term mortality risk in sepsis patients. These findings highlight the potential of antioxidant-based treatments as a promising approach in the management of sepsis [119].

### Mitochondria-induced apoptosis and its impact on sepsis progression

Autophagy is a cellular response to nutrient depletion or stress. It enables the recycling of cellular components, producing amino acids and fatty acids. These substances can then be metabolized and used in OXPHOS [120]. Autophagy is essential for recovering from critical illness. However, when mitochondria are dysfunctional, they may generate excessive ROS. This increase in ROS stimulates autophagy. Paradoxically, overly active autophagy can eventually lead to apoptosis [121]. During sepsis, increased levels of ROS and pro-inflammatory cytokines lead to an increase in mitochondrial membrane permeability. This permeability enhancement facilitates the release of apoptogenic factors into the cytoplasm, thereby activating the intrinsic apoptotic pathway [122]. The presence of dysfunctional and damaged mitochondria results in excessive production of ROS and insufficient energy supply. Mitophagy serves as a cellular mechanism to sequester and eliminate these impaired organelles, thereby mitigating such detrimental effects [123].

**Fig. 5.**
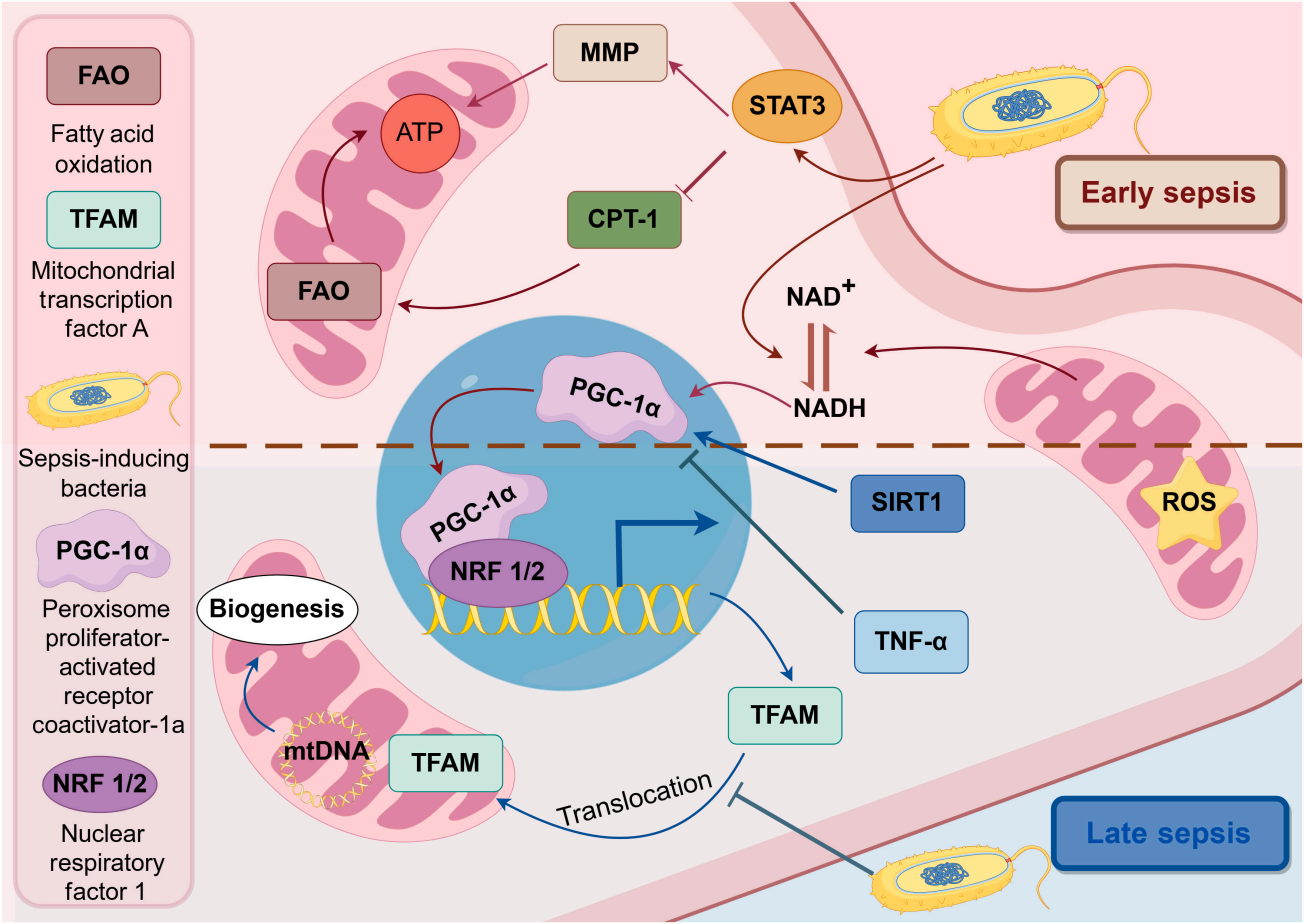
Sepsis and mitochondrial quality control mechanisms. Mitochondria, the powerhouses of the cell, rely on tightly regulated mechanisms to maintain their function. Fatty acid oxidation (FAO), a critical energy-generating pathway in mitochondria, is inhibited during sepsis to lead to insufficient energy production. Mitochondrial transcription factor A (TFAM), which stabilizes and regulates mitochondrial DNA transcription, is essential for mitochondrial genome maintenance. However, in sepsis, TFAM expression and function may be impaired, negatively affecting mitochondrial biogenesis. Additionally, nuclear respiratory factor (NRF), a key transcription factor for mitochondrial biogenesis, is activated by PGC-1α, a master regulator of energy metabolism. PGC-1α enhances NRF activity, promoting mitochondrial biogenesis and OXPHOS in response to energy demands. During sepsis, reduced PGC-1α expression and activity lead to diminished mitochondrial biogenesis, which worsens mitochondrial dysfunction and cellular impairment. (By Figdraw.)

Mitochondrial damage induces lymphocyte apoptosis, a characteristic feature of sepsis-induced immunosuppression, while NF-κB has been shown to promote mitophagy through the up-regulation of the autophagy receptor p62. This works as a self-regulatory mechanism to mitigate excessive inflammation and preserve homeostasis [124]. The mechanistic diagram illustrating the impact of mitochondrial injury on energy supply during sepsis is presented (Fig. 5).

In summary, mitochondrial dysfunction is key to sepsis pathophysiology, causing energy failure, oxidative stress, and cell death. Grasping these mechanisms is vital for creating therapies to maintain mitochondrial function, reduce inflammation, and enhance clinical outcomes. Future research ought to prioritize the identification of specific molecular targets within mitochondria that are optimally suited for therapeutic interventions. Additionally, efforts should be directed toward developing strategies that can be rapidly translated into clinical practice to benefit patients suffering from sepsis.

Mitochondrial dysfunction plays a crucial role in the development of sepsis, affecting not only cellular energy supply and apoptosis but also being closely related to metabolic changes in sepsis. Metabolic reprogramming is a characteristic of sepsis, interacting with mitochondrial dysfunction to collectively influence the progression of sepsis. The following section will provide a detailed exploration of the multifaceted aspects of metabolic reprogramming in sepsis.

## Metabolic Reprogramming in Sepsis

Metabolic reprogramming refers to the process by which cells, under specific physiological or pathological conditions, adjust their metabolic pathways and the utilization of metabolites to adapt to environmental changes or achieve certain functions. This process encompasses a variety of metabolic pathways, such as glycolysis, OXPHOS, fatty acid metabolism, and amino acid metabolism. Moreover, metabolic reprogramming also involves the redistribution of metabolic intermediates and the regulation of metabolite signaling pathways [125]. Metabolic reprogramming is also an important feature of sepsis. For example, during the hyperinflammatory phase of sepsis, cells preferentially switch to glycolysis to produce energy, even in the presence of oxygen, rather than relying on the more efficient OXPHOS process [126]. It is primarily driven by the down-regulation of OXPHOS due to mitochondrial dysfunction and the up-regulation of glycolysis resulting from the enhanced activation of the glycolytic pathway [127]. In the context of sepsis, metabolic reprogramming can be facilitated through the strategic targeting of mitochondrial quality control mechanisms, specifically modulating mitophagy and mitochondrial biogenesis, which actively contribute to the regulation of immune responses [109]. It has been proposed that sepsis-induced MOF is an adaptive response to enhance cell survival during infection. Mitochondrial dysfunction reduces ATP production efficiency, leading to insufficient energy in the affected organ, causing the body to enter a “dormant” state by down-regulating nonessential functions [128]. Consequently, metabolic reprogramming and energy deficit are considered fundamental triggers in the pathogenesis of MOF in sepsis.

Understanding key signaling pathways such as PI3K/Akt/mechanistic target of rapamycin (mTOR) and HIF-1α’s impact on glycolysis in metabolic reprogramming offers insights for therapeutic strategies to restore metabolic balance and improve patient outcomes [129]. For instance, inhibitors of aerobic glycolysis, such as 2-deoxy-d-glucose (2-DG), have been shown to attenuate glycolysis, leading to decreased systemic inflammation and kidney injury through the promotion of lactate/Sirtuin 3/AMPK-regulated autophagy. This suggests that 2-DG might be useful in therapeutic interventions [130]. Research data indicate that effective mitochondrial interventions are crucial for treating critically ill sepsis patients, and enhancing mitochondrial function may offer a promising treatment avenue [131]. This can be achieved by inducing mitochondrial biogenesis and enhancing antioxidant defenses. Maintenance of mitochondrial homeostasis will facilitate an early response to the disease in sepsis patients and improve patient survival [132].

### PI3K/Akt/mTOR signaling pathway

The PI3K/Akt/mTOR signaling pathway participates in the regulation of cellular metabolism, growth, and survival [133]. In sepsis, the PI3K/Akt/mTOR signaling pathway drives immune cells to undergo metabolic reprogramming. Specifically, this pathway mediates the down-regulation of apoptosis and autophagy. As a result, it reduces myocardial injury associated with sepsis [134]. In sepsis, the PI3K/Akt/mTOR pathway not only influences inflammatory and immune responses but also alters immune cell metabolism. It enhances glucose uptake and glycolysis to produce ATP, which is essential for immune cell activation and function [135]. This pathway is key for the normal immune function of neutrophils. Inhibition of the PI3K in murine models of sepsis has been associated with a marked reduction in neutrophil oxidative and phagocytic activities in vivo [136]. Although the transition to glycolysis is crucial for an effective immune response, it may also contribute to metabolic disturbances in sepsis, such as hyperglycemia and insulin resistance [137]. In the context of sepsis, the inhibition of mTOR has been shown to facilitate the clearance of damaged organelles, mitigating cellular stress, and enhancing survival [138]. This finding suggests that when formulating therapeutic strategies for the PI3K/Akt/mTOR pathway, it is necessary to carefully balance the enhancement of immune cell function and the preservation of the autophagic process. Only in this way can we optimize the treatment outcomes for sepsis patients.

Targeting the PI3K/Akt/mTOR pathway emerges as a promising therapeutic approach to modulate the immune response and improve organ protection in sepsis [139]. PI3K and mTOR inhibitors, including rapamycin, have been investigated for their capacity to attenuate excessive immune activation and enhance autophagy [140]. However, we must carefully consider the application of these therapeutic agents. This is because excessive suppression may impair fundamental immune functions and even exacerbate immunosuppression [141]. For instance, in murine models of sepsis, hyperactivation of the mTOR signaling pathway has been shown to induce pyroptosis in CD4^+^T cells, exacerbating immunosuppression associated with sepsis [142]. Therefore, additional research is required to ascertain the exact therapeutic window and to formulate strategies for the selective modulation of this pathway, with the aim of attaining an advantageous balance in metabolic and immune functions.

### HIF-1α and metabolic reprogramming

HIF-1α orchestrates the transition from OXPHOS to glycolysis in response to hypoxic conditions, frequently observed in septic tissues [93]. This intracellular metabolic reprogramming is indicative of the Warburg effect, previously discussed, wherein immune cells are capable of rapidly generating energy, even under hypoxia, to sustain their immune functions [143]. In sepsis, the glycolytic pathway directly causes elevated lactate production. This increase in lactate is linked to metabolic acidosis and ultimately results in organ dysfunction [144].

The up-regulation of glycolytic activity mediated by HIF-1α in sepsis not only fulfills the immediate energy demands of immune cells but also has the capacity to influence the overall systemic metabolic milieu [145]. Nevertheless, increased glycolysis may result in metabolic exhaustion, thereby diminishing the responsiveness and efficacy of immune cells, which contributes to the immunosuppressive phase of sepsis [146]. NETosis is a unique type of cell death in neutrophils, different from apoptosis and necrosis. It is marked by heightened glycolytic activity, which helps form neutrophil extracellular traps (NETs). These NETs can capture and eliminate harmful pathogens, and this entire process is what we call NETosis [147].

Modulating the activity of HIF-1α presents a promising therapeutic strategy for sepsis. Inhibition of HIF-1α can mitigate pathological glycolytic translocation and its related metabolic complications, thereby reducing tissue damage and enhancing organ function [148]. Considering the critical role of HIF-1α in sustaining immune cell function under hypoxic conditions, such interventions must be meticulously calibrated to prevent impairing the host’s capacity to combat infections [149]. Combining the regulation of HIF-1α with other metabolic treatments, like improving mitochondrial function, could provide a more effective treatment approach for sepsis [150].

In conclusion, metabolic reprogramming is a crucial component of sepsis, exerting a profound impact on disease progression and patient prognosis. By targeting key pathways such as PI3K/Akt/mTOR and HIF-1α, and integrating the role of the microbiome, more effective and personalized treatment strategies can be formulated to address the complex metabolic challenges associated with sepsis. In the future, it is possible to deeply analyze the molecular mechanisms of metabolic reprogramming in immune cells and systematically elucidate the changes in metabolic phenotypes of different immune cell subsets during sepsis, providing a theoretical basis for innovative treatment strategies. Researchers can also commit to conducting large-scale, multi-center clinical trials to validate the findings of basic research and explore new biomarkers. Meanwhile, the use of advanced technologies such as machine learning to analyze patients’ clinical data may help identify heterogeneous phenotypes of sepsis and promote the development of precision medicine. In addition, by strengthening the cooperation between basic research and clinical practice, the development and clinical application of new drugs and treatment strategies can be promoted, and the therapeutic effect of sepsis can be improved. With a deeper understanding of the mechanisms of sepsis, more effective preventive and treatment strategies are expected to be achieved in the future, fundamentally improving the prognosis of sepsis patients.

## The Prospect of Targeted Therapy

Current limitations in sepsis management have highlighted the critical need to investigate the underlying signaling pathways involved in its pathophysiology, offering potential avenues for the development of more effective interventions. Sepsis is characterized by a dysregulation of systemic immunity, encompassing both anti-inflammatory and pro-inflammatory responses. Targeting signaling pathways, including JAK/STAT and PI3K/Akt, has the potential to modulate immune cell function, restore immune equilibrium, and suppress excessive inflammatory responses [151]. Furthermore, sepsis can result in MODS. By modulating specific signaling pathways such as HIF-1α and AMPK, it is possible to enhance organ perfusion and reduce organ damage [152].

Numerous challenges remain before research on sepsis can be effectively translated into clinically viable treatment options. From a pathogenesis perspective, sepsis encompasses multiple signaling pathways that interact to establish a complex regulatory network [153]. Therefore, identifying appropriate key signaling pathways and intervention nodes presents a major challenge. From the perspective of patient treatment, targeted therapy must account for individual variability, as there are distinct differences in the etiology, clinical condition, and immune status among patients with sepsis. These differences lead to varying degrees of signaling pathway activation. Despite the increasingly identified drugs with potential efficacy in treating sepsis, the development of clinical therapeutics for human application remains challenging. The process of drug development targeting signaling pathways must address issues such as drug selectivity, bioavailability, and toxic side effects. Furthermore, it is inherently complex and costly [154]. In the context of clinical trials for sepsis, the progression of the disease is highly variable. Careful consideration of numerous factors in the trial design, including sample size, timing of intervention, and methods for evaluating efficacy, is needed to ensure the reliability of the results [155]. There have been advancements in research on agents targeting signaling pathways for sepsis treatment (Table 3).

**Table 3.** Therapeutic agents and impacts on sepsis-related signaling pathways

Signaling pathway	Pharmacological interventions	Mechanism	Use in the context of sepsis	Effects	References
	3PO (PFKFB3 inhibitor)	3PO inhibits PFKFB3, a glycolytic activator, thereby suppressing LPS-induced NF-κB-p65 nuclear translocation, reducing endothelial cell glycolysis, inflammation, and permeability, and alleviating ALI.	It is utilized to inhibit the activity of the NF-κB signaling pathway, attenuating the inflammatory response associated with sepsis and treating ALI induced by sepsis.	It attenuates the inflammatory response of the vascular endothelium and confers protection to mice against LPS-induced ALI.	[184]
	Angiotensin-(1–7)	Angiotensin-(1–7) inhibits the NF-κB and MAPK signaling pathways, diminishes ROS production, mitigates oxidative stress, and alleviates inflammatory responses. It ameliorates mitochondrial structure and function in cardiomyocytes and suppresses cardiomyocyte apoptosis.	Regulate the balance of the RAAS and decrease Ang-II levels; sustain the mitochondrial energy supply in cardiomyocytes, inhibit apoptosis in these cells, and mitigate myocardial damage.	By reducing myocardial tissue inflammation, inhibiting cellular infiltration, and alleviating oxidative stress, the severity of SIC can be decreased.	[185]
	Curcumin	Curcumin inhibits the activation of the JAK2/STAT3 signaling pathway, suppressing the expression of downstream inflammatory mediators and mitigating tissue damage associated with sepsis.	Curcumin exerted a protective effect against sepsis-induced kidney injury, attenuated inflammatory responses and apoptotic processes, and improved renal function parameters.	Decrease serum concentrations of creatinine (Cr), blood urea nitrogen (BUN), and cystatin C (Cys-C) in murine models of sepsis.	[186]
Wnt/β-catenin	HOXA cluster antisense RNA 2 (HOXA-AS2) analogs or agonists	The up-regulation of long noncoding RNA HOXA-AS2 can inhibit the Wnt/β-catenin and NF-κB signaling pathways, thereby reducing damage to renal tubular epithelial cells.	It can be used in treating AKI induced by sepsis.	It can mitigate damage to renal tubular epithelial cells and preserve kidney function while reducing inflammation and enhancing sepsis patients’ survival.	[187]
	siRNA or β-catenin inhibitors	β-catenin can drive the activation of the NLRP3 inflammasome, while its inhibitors reduce the interaction between NLRP3 and ASC by blocking this mechanism, thereby alleviating the inflammatory response.	In the treatment of sepsis patients, particularly those exhibiting an excessive inflammatory response, β-catenin inhibitors have been shown to attenuate LPS-induced systemic inflammation.	β-Catenin inhibitors can mitigate LPS-induced systemic inflammation.	[188]
Notch	Dual antiplatelet therapy (DAPT)	DAPT, a Notch signaling pathway inhibitor, can suppress ACE activity, reduce Ang-II production, and alleviate damage to vascular endothelial cells.	It can mitigate vascular endothelial cell damage and ameliorate endothelial dysfunction induced by sepsis.	DAPT can alleviate damage to pulmonary vascular endothelial cells by inhibiting Ang-II production, thereby exerting a protective effect against sepsis-induced lung injury.	[189]
PI3K/Akt	Huanglian Jiedu decoction (a traditional herbal formulation)	Quercetin, the main active ingredient in Huanglian Jiedu decoction, can reduce ROS levels in the lung tissue of septic rats, decrease HMGB1 expression, and inhibit the activation of the PI3K/Akt pathway, thereby protecting against sepsis-induced lung injury.	Huanglian Jiedu decoction is used in clinical practice for the treatment of sepsis, demonstrating notable therapeutic efficacy.	It can reduce inflammation, reduce organ damage, and improve survival in patients with sepsis.	[190]
TGF-β	Dexmedetomidine	Dexmedetomidine prevents TGF-β1-induced epithelial–mesenchymal transition (EMT) by inhibiting the phosphorylation of the JNK signaling pathway.	Dexmedetomidine alleviates LPS-induced renal injury by inhibiting myofibroblast activation, NLRP3 inflammasome formation, and cell necrosis, suppressing EMT, and reducing renal fibrosis.	Dexmedetomidine mitigates renal fibrosis and safeguards renal tubular epithelial cells from damage by inhibiting the EMT process, enhancing renal function in patients with sepsis.	[191]
MAPK	Fisetin	Fisetin has been shown to mitigate renal inflammation and apoptosis, preserving organ function in murine models of septic AKI. This protective effect is achieved through the inhibition of the Src-mediated NF-κB p65 and MAPK signaling pathways.	Fisetin can mitigate renal inflammation and apoptosis, enhancing survival of mice with LPS-induced septic AKI.	It can decrease inflammation and tissue damage in septic animals, enhancing survival. Fisetin might also weaken the immune response, raising the risk of infection.	[192]
TLR	Berberine	Berberine binds to the TLR4/MD-2 receptor and inhibits LPS-induced activation of the TLR4 signaling pathway.	Berberine can reduce LPS-induced intestinal permeability increase and inflammatory response, and alleviate sepsis-induced MODS.	Berberine can inhibit the production of inflammatory mediators, protect the intestinal barrier, reduce lung and myocardial injury, and decrease the mortality rate in sepsis models.	[193]
	Nano-curcumin	Nano-curcumin alleviates sepsis-related inflammatory responses by inhibiting the TLR4 signaling pathway.	The prognosis of patients with severe sepsis in the intensive care unit may be enhanced.	Nano-curcumin supplementation has the potential to attenuate inflammatory biomarkers and oxidative stress indices, as well as enhance endothelial function in critically ill patients with sepsis.	[194]
Autophagy and apoptosis	Emodin	Emodin facilitates the activation of the BDNF/TrkB signaling pathway, enhancing autophagy, inhibiting apoptosis, and mitigating inflammatory responses.	Enhances cognitive function and mitigates hippocampal neuronal damage. This process not only reduces inflammation, inhibits apoptosis, and promotes autophagy but also facilitates cellular repair and improves organ function.	The application of emodin in the management of sepsis predominantly targets SAE.	[195]
	Quercetin	Quercetin, as a SIRT1 deacetylase activator, promotes the deacetylation of p53, which helps its nuclear retention and activation of autophagy. This mechanism contributes to alleviating sepsis-related acute kidney injury.	Quercetin alleviates renal injury in CLP-induced septic mice, manifested as reduced levels of renal injury biomarkers including kidney injury molecule-1 and Scr, and improved renal function.	Quercetin exhibits limited bioavailability, which requires elevated dosages to attain therapeutic efficacy.	[196]
Pyroptosis	Diulfiram	Disulfiram can inhibit gasdermin D pore formation, down-modulating pyroptosis and the release of inflammatory factors.	Disulfiram is approved for the treatment of alcohol dependence and is anticipated to enhance the prognosis of sepsis by inhibiting the pyroptosis signaling pathway.	In a mouse sepsis model, disulfide effectively reduced mortality and decreased the levels of inflammatory cytokines such as IL-1β, TNF, and IL-6 in serum.	[197]
	Buformin	Buformin can enhance autophagy and up-regulate Nrf2 protein levels via AMPK-dependent pathways. This modulation inhibits the NLRP3 inflammasome, preventing pyroptosis and mitigating lung inflammation and tissue damage.	When evaluating the efficacy and safety of buformin in sepsis, rigorous clinical trials are still required.	It inhibits the expression of NLRP3-related proteins and alleviates sepsis-induced ALI.	[198]
Ferroptosis	Uridine	Uridine activates the Nrf2 pathway, promotes the expression of antioxidant genes, and reduces ACSL4, thereby helping to prevent ferroptosis, alleviate inflammatory responses, and mitigate oxidative stress.	Uridine can mitigate sepsis-induced ALI and ameliorate lung damage, inflammatory responses, tissue iron levels, and lipid peroxidation.	Uridine is a natural substance. It affects multiple signaling pathways for various therapeutic benefits.	[199]

Sepsis is a complex disease involving multiple biological pathways, and targeted therapy has become a key strategy to optimize its management. Bioinformatics technologies have demonstrated substantial application value in the exploration of targeted therapies for sepsis. For instance, a study using network pharmacology analysis found that heparin may act on multiple targets related to immune regulation and coagulation function, providing a new perspective for targeted sepsis therapy. Further molecular dynamics simulations validated the binding stability and affinity of heparin with these targets, laying a theoretical foundation for the development of heparin-based targeted treatment strategies [156]. Furthermore, individualized treatment can be achieved by intervening in specific signaling pathways and combining immunomodulation, metabolic regulation, nanotechnology, mAbs, and microRNAs, thereby enhancing therapeutic efficacy and reducing side effects [157]. In the field of immunomodulatory therapy, cytokine-targeted therapy has attracted significant attention. Additionally, metabolic target therapy has gradually become an important direction in sepsis management. Sepsis patients exhibit marked metabolic disorders, and targeted treatment of hypoxemia can reduce ROS accumulation and improve outcomes [158]. The dual role of the calcium-sensing receptor (CaSR) in sepsis has also drawn attention: Its activation can both alleviate organ dysfunction and potentially exacerbate inflammatory responses. In-depth analysis of the CaSR signaling pathway will provide new insights for targeted therapy [159]. Therapeutic strategies targeting metabolic endpoints not only improve clinical outcomes in sepsis but also hold promise for future treatment directions. Lipid nanoparticles, a nanosystem composed of lipid molecules, have demonstrated advantages in sepsis treatment due to their excellent drug encapsulation and cell-targeting properties [160]. Nanomedicine design offers a new perspective for sepsis treatment by regulating the function of immune cells. Different nanoparticles can induce immune cells to differentiate in specific directions. Nanodrug design targeting immune cells shows broad prospects in both the treatment and early diagnosis of sepsis. Meanwhile, the application of nanotechnology in early diagnosis may also transform the management paradigm of sepsis. mAb therapy has also achieved notable efficacy in sepsis. mAbs targeting LPS and related inflammatory signaling pathways can neutralize the pathogenic effects of LPS and inhibit inflammation [161]. Moreover, research suggests that modulating the activation status of immune cells, particularly through targeted therapy for CD38-highly expressed monocytes, can mitigate sepsis-induced inflammation and enhance patient survival [162].

Targeted therapy for sepsis has made remarkable progress over the past few years, with these emerging approaches providing new perspectives and possibilities for improving sepsis diagnosis and treatment. However, despite the potential benefits of these therapeutic strategies, caution is still required in clinical application, particularly in balancing perspectives and findings across different studies. Future efforts may focus on the discovery of new targets and the exploration of multi-target combination therapy strategies. Sepsis is not caused by a single etiology but rather involves multiple biological pathways; thus, identifying new therapeutic targets and adopting multi-target combination therapy will be important directions for future research. By integrating diverse treatment modalities, future sepsis therapy will become more precise, tailored to individual patient needs, enhancing treatment efficacy and reducing mortality. This requires not only continuous advancements in basic research but also clinical trial validation to verify the effectiveness and safety of these new strategies, ultimately offering new possibilities for improving patient outcomes.

## Future Directions and Conclusion

Sepsis represents a major global health challenge. It is characterized by dysregulated immune response, metabolic disturbances, and organ dysfunction, and is associated with high morbidity and mortality rates. Thus, there is an urgent need to develop novel and more efficacious therapeutic interventions. Despite ongoing research efforts, substantial challenges persist within the existing literature regarding the understanding of sepsis pathophysiology and the identification of effective targets for treatment strategies.

Although substantial progress has been made in studying sepsis signaling pathways, we still lack a deep understanding of how these pathways interact with each other and what specific roles they play at different pathological stages. Thus, future research should examine the alterations in various signaling pathways and evaluate their specific roles at each stage of sepsis progression. This approach will facilitate the precise identification of intervention windows and the development of targeted therapeutic strategies. This includes the potential for timely modulation of these pathways within the immune response to sepsis, ideally achieving control and resolution of systemic infections prior to the onset of a cytokine storm.

A major challenge in sepsis research is the translation of knowledge acquired using preclinical models into clinical practice. Genetic factors, individual variability, and disparities in immune system backgrounds greatly influence the progression and prognosis of sepsis in patients, thereby complicating the application of a uniform treatment regimen. Insufficient human studies on sepsis have been conducted. Therefore, there is an urgent need for personalized therapeutic strategies in the diagnosis and management of sepsis. Future research should give priority to identifying and screening sepsis-related biomarkers and developing personalized intervention strategies. This will help improve the specificity and effectiveness of treatment methods. Current treatment models often ignore the heterogeneity of sepsis. They tend to use a standardized treatment approach, which may not work well for all patients due to individual differences. Future efforts should focus on the identification of specific markers within the signaling pathway, enabling the stratification of patients according to their unique pathophysiological characteristics. These biomarkers have the potential to inform personalized treatment strategies, facilitate the early identification of high-risk sepsis patients, and promote timely interventions. This approach would allow clinicians to tailor therapeutic interventions to address specific dysregulations in signaling pathways or distinct metabolic profiles.

In summary, a thorough understanding of the signaling pathways in sepsis is crucial for developing effective therapeutic strategies. Future research can focus on several key areas: first, elucidating the complex interactions between sepsis-related signaling pathways; second, developing innovative combination therapies targeting multiple signaling pathways; and third, establishing novel immune monitoring indicators. A comprehensive understanding of the intricate signaling pathway network in sepsis is essential for identifying new targets that have the potential to improve patient outcomes. Despite the progress made in sepsis research, many challenges remain. Advancing in these research directions holds promise for bringing new breakthroughs to the clinical treatment of sepsis, thereby improving patient survival rates and quality of life.

## Data Availability

Data sharing is not applicable to this article as no datasets were generated or analyzed during the current study. All information is derived from publicly available articles and datasets.
